# 血液肿瘤患者回输嵌合抗原受体T细胞后28天内感染病原学和临床特征分析

**DOI:** 10.3760/cma.j.issn.0253-2727.2021.09.005

**Published:** 2021-09

**Authors:** 英楠 李, 孟仪 都, 成功 李, 寅嫱 张, 文静 雒, 海明 寇, 恒 梅, 豫 胡

**Affiliations:** 华中科技大学同济医学院附属协和医院血液科，湖北省肿瘤疾病细胞治疗临床医学研究中心，武汉 430020 Institute of Hematology, Union Hospital, Tongji Medical College, Huazhong University of Science and Technology; Hubei Clinical Medical Center of Cell Therapy for Neoplastic Disease, Wuhan 430022, China

**Keywords:** 嵌合抗原受体T细胞免疫治疗, 血液肿瘤, 感染, 临床分析, Chimeric antigen receptor T cell, Hematological malignancy, Infection, Clinical characteristics

## Abstract

**目的:**

探讨血液肿瘤患者在回输嵌合抗原受体T细胞（CAR-T细胞）后28 d内感染的发生率、临床和微生物学特征及危险因素。

**方法:**

回顾性分析2016年1月至2020年12月在华中科技大学同济医学院附属协和医院血液科成功接受CAR-T细胞输注的170例血液肿瘤患者基线资料以及回输后28 d内发生感染的临床与病原菌特征。使用泊松回归评估基线特征与感染密度的相关性，单因素和多因素Cox比例风险回归模型评估CAR-T细胞回输后感染的高危因素。

**结果:**

170例患者中急性淋巴细胞白血病（ALL）72例、非霍奇金淋巴瘤（NHL）56例、多发性骨髓瘤（MM）42例，99例患者在28 d内共发生119次感染，累积感染发生率为（58.2±3.8）％；其中细菌感染98次（45.9％），总感染密度为2.01，首次感染发生的中位时间为回输后12（1～18）d。多因素泊松回归显示ALL患者、难治性疾病、既往30 d内感染病史、回输前ANC<0.5×10^9^/L、前期治疗方案≥4种的患者在28 d内与感染密度有更高的相关性；多因素Cox回归显示≥3级细胞因子释放综合征（CRS）是发生感染的独立危险因素（*HR*＝3.74，95％*CI* 1.29～5.72，*P*<0.001）。

**结论:**

感染是血液病患者CAR-T治疗后常见的并发症之一，不同肿瘤的感染发生率与类型有所差异，但均以细菌感染为主。ALL患者、难治性疾病、既往30天内感染病史、回输前ANC<0.5×10^9^/L、前期治疗方案≥4种与感染密度相关；3级以上CRS是感染发生的独立危险因素。**中国临床试验注册中心：**ChiCTR-OIC-17011180、ChiCTR1800018143

## Abstract (en)

**Objective:**

To explore the incidence, clinical and microbiological characteristics and risk factors of infection in patients with acute lymphoblastic (ALL), non-Hodgkin lymphoma (NHL), and multiple myeloma (MM) within 28 days after CAR-T cell infusion. It provides data support for early identification of infection and the rational use of antibacterial drugs in these patients.

**Methods:**

We retrospectively analyzed the baseline data of 170 patients with ALL, NHL and MM who received chimeric antigen receptor-modified T (CAR-T)-cell treatment in the Department of Hematology of Wuhan Union Hospital from January 2016 to December 2020, and the clinical characteristics of infection within 28 days after infusion, including 72 patients with ALL, 56 patients with NHL, and 42 patients with MM; we used Poisson regression and Cox proportional hazard regression models to assess high-risk factors for infection before and after infusion, respectively.

**Results:**

Among 170 patients, 119 infections occurred in 99 patients within 28 days, with a cumulative infection rate of 58.2％. Seventy-eight patients had 98 bacterial infections and the cumulative incidence of bacterial infection was 45.9％. The infection density was 2.01, and the median time for the first infection was about 12 days after infusion. The adjusted baseline characteristic model showed that ALL patients, previous 30 days of infection history, refractory disease, absolute neutrophil count (ANC)<0.5×10^9^/L before infusion and ≥4 prior antitumor treatment regimens had a higher infection density within 28 days; grade 3 or 4 CRS was the only high-risk factor related to infection after infusion in the multivariate analysis.

**Conclusion:**

Infection is a common complication of CAR-T cell therapy in patients with hematologic malignancy. Bacterial infections occur in most patients regardless of the type of disease. ALL patients, previous 30 days of infection history, refractory disease, ANC<0.5 × 10^9^/L before infusion and grade 3 or 4 CRS are risk factors for infection. **Chinese Clinical Trial Register**: ChiCTR-OIC-17011180, ChiCTR1800018143

嵌合抗原受体T细胞（CAR-T细胞）疗法作为一种新兴的细胞免疫治疗方法，在难治复发血液系统肿瘤患者中疗效显著[1]–[4]，感染与CAR-T细胞治疗的安全性密切相关，国外研究发现CAR-T细胞治疗后危及生命感染发生率为1.98％～6％[5]–[6]，但未明确提出如何早期识别极重度感染以及有效的预防措施。国内关于CAR-T细胞治疗后早期感染的研究相对较少，尤其关于感染发生率、类型、早期识别以及抗菌药物使用等数据并不充分。因此，本研究我们分析接受CAR-T细胞治疗的170例血液肿瘤患者在CAR-T回输后28 d内发生感染的流行病学特征，并筛选感染的高危因素，为早期识别高危患者提供依据，从而降低重度甚至危及生命感染的发生率。

## 病例与方法

一、病例资料

回顾性分析2016年1月至2020年12月华中科技大学同济医学院附属协和医院血液科CAR-T临床试验（中国临床试验注册中心：ChiCTR-OIC-17011180、ChiCTR1800018143）入组的170例患者。纳入标准：①罹患血液系统肿瘤性疾病；②满足CAR-T临床试验入组标准；③随访观察28 d或发生死亡事件。排除标准：①遵循CAR-T临床试验排除标准；②排除未治愈的活动性感染者；③排除活动性乙型肝炎或丙型肝炎病毒感染者。研究方案经华中科技大学同济医学院附属协和医院伦理委员会批准（伦理号：2016.002-02、2018-005），所有患者均充分知情并签署知情同意书。

二、研究方案

所有资料通过病历检索和临床试验观察表（CRF表）完成采集，记录项目包括患者人口学特征、诊断、治疗方法、危险因素、临床特征、微生物学特征、抗感染治疗方案及生存情况。本研究为观察性研究，所有患者的诊治均由临床医师判断，并按照临床常规处理方案执行。同一患者回输CAR-T后住院期间发生多次感染，被记录为多个例次；如果患者于回输后28 d内未出现感染，则观察至出院为止；如果患者于回输后出现感染，则观察至抗感染治疗结束或患者死亡。所有患者在回输前均接受淋巴耗竭化疗方案，急性髓系白血病（ALL）和非霍奇金淋巴瘤（NHL）患者具体方案为：环磷酰胺750 mg·m^−2^·d^−1^，−7～−6 d；氟达拉滨30 mg·m^−2^·d^−1^，−7～−5 d；多发性骨髓瘤（MM）患者具体方案为：环磷酰胺250 mg·m^−2^·d^−1^，−5～−3 d；氟达拉滨25 mg·m^−2^·d^−1^，−5～−3 d。101例患者回输剂量为2×10^6^/kg，69例回输剂量为4×10^6^/kg；回输CAR-T细胞后监测相关指标，包括体温、血氧饱和度、血压、心率、血常规、肝肾功能、电解质、细胞因子等，同时评估细胞因子释放综合征（CRS）、免疫效应细胞相关神经毒性综合征（ICANS）等CAR-T相关不良反应并及时处理，处理方案遵循文献[7]；血清IL-6使用流式细胞术检测。

三、定义

感染的诊断参照文献[8]标准，临床诊断及病原学诊断的感染均纳入分析，特定部位感染通过具有红、肿、热、痛炎症反应等临床表现或影像学、实验室检查等手段判断。感染密度计算方式为28 d内的感染人数/总人天数×100（每100天内发生感染的人数）；粒细胞缺乏（粒缺）定义为ANC<0.5×10^9^/L或预计未来48 h内ANC<0.5×10^9^/L[9]；感染严重程度遵照CTCAE（4.0.3版本）进行判断[5],[10]；CRS定义为一种由细胞因子释放引起的以发热、呼吸急促、头痛、心动过速、低血压、皮疹和（或）缺氧为特征的疾病，CRS分级按照美国移植和细胞治疗学会（ASTCT）专家共识[11]来进行评估。

1. 细菌感染分为血流感染和特定部位感染：①血流感染：同一天血培养检出不同病原体为多个例次感染，不同时间血培养检出相同病原体则需要与前次间隔足够长的时间且经过治疗后血培养转阴才可被认为多个例次的感染；单独一次血培养提示皮肤常见菌（如类白喉杆菌、非致病分枝杆菌或凝固酶阴性的葡萄球菌）且没有全身症状或全身使用抗生素则不算做一次感染。②特定部位感染：无菌部位微生物培养阳性或非无菌部位存在感染的临床表现且影像学阳性和抗感染治疗有效。

2. 病毒感染分为呼吸道病毒和其他：①呼吸道病毒：鼻咽拭子或痰培养病毒阳性、PCR检测阳性、支气管肺泡灌洗中的呼吸道病毒阳性，或明显的呼吸道症状、影像学提示有新的或变化的肺浸润且排除细菌感染。②其他：包括血标本提示暴发性乙型肝炎病毒（HBV）（入组前患者HBV DNA定量为阴性，回输后HBV DNA定量大于1×10^3^拷贝/ml）、疱疹病毒、巨细胞病毒和EB病毒等。

3. 真菌感染则依据血培养阳性并按照2008侵袭性真菌病诊断标准[12]。

四、数据收集及统计学处理

感染密度计算为每100个患者日的平均感染数。为了确定和评估与感染密度相关的基线特征和感染的高危因素，使用单因素和逐步多因素泊松回归和Cox比例风险回归分析，单因素*P*<0.25的变量纳入多因素分析。采用Stata 15和Prism 8进行统计分析以及作图。所有比较均为双侧检验，*P*<0.05为差异有统计学意义。

## 结果

1. 患者基本信息和治疗特征：170例接受CAR-T的血液病患者基线特征见表1，中位年龄41（10～72）岁，女性69例（40.6％），ALL 72例、NHL 56例、MM 42例。基础疾病情况：ALL患者中，合并糖尿病3例，合并支气管哮喘、高血压各1例；NHL患者中，1例既往冠状动脉粥样硬化性心脏病史、经皮冠状动脉介入治疗（PCI）术后和糖尿病史，乳腺癌康复1例、合并高血压1例；MM患者中，合并高血压4例，1例既往脑梗死病史，患者入组前基础疾病病情稳定。29例（40.3％）ALL患者、7例（12.5％）NHL患者和11例（26.2％）MM患者在既往30 d内发生过感染事件；回输前患者的中位前期治疗方案为4（3～7）种，27例（15.9％）患者曾接受自体或异基因造血干细胞移植；72例ALL患者中有57例（79.2％）为复发难治；淋巴耗竭化疗方案前，21例（21％）ALL患者IgG<4 g/L，84例（49.4％）患者淋巴细胞绝对值计数（ALC）<0.2×10^9^/L，33例（19.4％）患者ANC<0.5×10^9^/L。回输后，在所有发生感染的99例患者中，中位粒缺时间为12（0～22）d，中性粒细胞恢复中位时间为18（3～37）d，79例（46.5％）患者白蛋白水平低于正常。38例（22.4％）患者发生了3级以上的CRS，10例（5.88％）患者发生了神经毒性，41例（24.1％）患者接受了糖皮质激素或托珠单抗的治疗，其中单用激素3例（1.76％）、单用托珠单抗18例（10.6％）、糖皮质激素联合托珠单抗20例（11.8％）。8例（4.71％）患者曾进入ICU治疗，包括6例（8.33％）ALL和2例（3.57％）NHL患者。

**表1:** 接受嵌合抗原受体T细胞（CAR-T细胞）治疗的血液病患者基本信息和治疗特征

基本信息和治疗特征	总体（170例）	ALL（72例）	NHL（56例）	MM（42例）
年龄［岁，*M*（范围）］	41（10～72）	29（10～65）	49（29～72）	65（39～72）
女性［例（％）］	69（40.6）	30（41.7）	20（35.7）	19（45.2）
基础疾病［例（％）］	13（7.65）	5（6.94）	3（5.36）	5（11.9）
前期治疗［例（％）］^a^	68（40.0）	29（40.3）	31（55.4）	8（19.0）
移植史［例（％）］	27（15.9）	15（20.8）	8（14.3）	4（9.52）
感染史［例（％）］^b^	47（27.6）	29（40.3）	7（12.5）	11（26.2）
化疗前IgG<4g/L［例（％）］	NA	21（29.2）	10（17.9）	NA
化疗前ALC<0.2×10^9^/L［例（％）］	84（49.4）	37（51.4）	30（53.6）	17（40.5）
化疗前ANC<0.5×10^9^/L［例（％）］	33（19.4）	19（26.4）	11（19.6）	3（7.1）
难治病例［例（％）］	135（79.4）	57（79.2）	47（83.9）	31（73.8）
回输CAR-T细胞量				
2×10^6^/kg［例（％）］	101（59.4）	50（69.4）	45（80.4）	6（14.3）
4×10^6^/kg［例（％）］	69（40.6）	22（30.6）	11（19.6）	36（85.7）
回输后特征				
粒缺持续时间［d，*M*（范围）］	12（0～22）	14（0～22）	5（0～9）	8（0～13）
ANC恢复时间［d，*M*（范围）］^c^	18（3～37）	18（3～37）	18（14～28）	20（14～30）
白蛋白<35g/L［例（％）］	79（46.5）	39（54.2）	21（37.5）	19（45.2）
CRS分级［例（％）］				
0～2级	132（77.6）	54（75.0）	45（80.4）	33（78.6）
3～4级	38（22.4）	18（25.0）	11（19.6）	9（21.4）
神经毒性［例（％）］	10（5.9）	5（6.9）	3（5.3）	2（4.8）
糖皮质激素/托珠单抗治疗［例（％）］	41（24.1）	21（29.2）	11（19.6）	9（21.4）
进入ICU治疗［例（％）］	8（4.7）	6（8.3）	2（3.6）	0（0.0）

注：ALL：急性淋巴细胞白血病；NHL：非霍奇金淋巴瘤；MM：多发性骨髓瘤；ALC：淋巴细胞绝对计数；CRS：细胞因子释放综合征；ICU：重症监护室。^a^前期治疗方案≥4种；^b^既往30 d内发生感染；^c^回输后至ANC第1次≥0.5×10^9^/L的时间；NA：不适用

2. 回输后28 d细菌、病毒、真菌感染的临床特征：见表2。在CAR-T细胞回输后的28 d内，170例患者中99例（58.2％）发生了119例次感染。共分析了4930个患者日，感染密度为2.01。首次感染发生的中位时间为12（1～18）d。有80％的首次感染发生在14 d内。总感染累积发生率（58.2±3.8）％，细菌感染（45.9±3.8）％，病毒感染（8.2±2.1）％，真菌感染（4.1±1.5）％。

**表2:** 回输CAR-T细胞后28 d内不同感染类型的感染例次数、感染例数及感染密度

感染类型	感染例次数（例次）				感染例数［例（％）］				感染密度			
	总体	ALL	NHL	MM	总体	ALL	NHL	MM	总体	ALL	NHL	MM
任何感染	119	61	29	29	99（58.2）	46（63.9）	29（51.8）	24（57.1）	2.01	2.20	1.79	1.97
细菌感染	98	51	21	26	78（45.9）	36（50.0）	21（37.5）	21（50.0）	1.58	1.72	1.29	1.72
血流感染	62	34	12	16	61（35.8）	34（47.2）	12（21.4）	15（35.7）	1.24	1.63	0.74	1.23
特定部位感染	36	17	9	10	34（20.0）	17（23.6）	9（16.1）	8（19.0）	0.69	0.81	0.55	0.66
病毒感染	14	6	6	2	14（8.2）	6（8.3）	6（10.7）	2（4.8）	0.28	0.29	0.37	0.16
呼吸道病毒感染	5	0	4	1	5（2.9）	0（0.0）	4（7.1）	1（2.4）	0.10	0	0.25	0.08
其他病毒感染	9	6	2	1	9（5.3）	6（8.3）	2（3.6）	1（2.4）	0.18	0.29	0.12	0.08
真菌感染	7	4	2	1	7（4.1）	4（5.6）	2（3.6）	1（2.4）	0.14	0.19	0.12	0.08

注：CAR-T细胞：嵌合抗原受体T细胞；ALL：急性淋巴细胞白血病；NHL：非霍奇金淋巴瘤；MM：多发性骨髓瘤

细菌感染最常见，78例（45.9％）患者发生了98次细菌感染事件。61例血流感染患者中16例（9.41％）明确病原体，其中14例（8.2％）为革兰阴性菌感染，2例（1.2％）为耐甲氧西林的凝固酶阴性葡萄球菌（MRSCN）感染。在36次特定部位感染事件中，肺部感染22次，检出嗜麦芽窄食单胞菌、大肠埃希菌、耐碳青霉烯类鲍曼不动杆菌（CRAB）各1株，余19次肺部感染病原体未明确。不同疾病类型分类的特定部位感染事件：ALL患者中，革兰阳性球菌皮肤软组织感染、革兰阳性球菌消化道感染、输液港局部感染各1例，病原体未明上呼吸道感染3例；NHL患者中包括3例消化道感染和2例上呼吸道感染；MM患者中包括2例消化道感染和1例急性腮腺炎。

病毒感染是第2常见的感染，14例（8.2％）患者发生了14次病毒感染事件。5例患者感染了呼吸道病毒，包括2例鼻病毒、2例甲型流感病毒和1例副流感病毒，其中2例患者出现了下呼吸道疾病。1例患者感染单纯疱疹病毒、2例患者感染带状疱疹病毒，4例患者发生了HBV。在2例发生4级CRS患者的血浆中分别检测出了EBV和巨细胞病毒（CMV），两例患者均未发生病毒导致的器官疾病。

真菌感染中，7例（4.12％）患者中发生了7次侵袭性霉菌感染事件，包括2例光滑念珠菌肺部感染，2例白色念珠菌肺部感染，2例光滑念珠菌血症和1例曲霉菌肺部感染。这7例患者均发生了3级以上的CRS或神经毒性，需要使用托珠单抗和（或）糖皮质激素治疗，其中5例曾接受自体或异基因造血干细胞移植。

不同肿瘤感染发生情况见表2，在72例ALL患者中，46例（63.9％）发生感染；在56例ALL患者中，29例（51.8％）发生感染，在42例MM患者中，24例（53.1％）发生感染。

不同肿瘤发生感染的严重程度见表3，在所有的感染事件中，7例（4.1％）患者发生了7次危及生命感染事件，包括4例ALL患者，1例NHL患者和2例MM患者，3例（1.76％）患者死亡。其中2例（1.18％）ALL患者死亡（1例曲霉菌肺部感染患者回输前存在基础感染并在回输后进一步发展导致死亡）。1例（0.59％）NHL患者发生了危及生命感染事件，该患者有重度CRS且伴有耐碳青霉烯类鲍曼不动杆菌肺部感染。CAR-T回输后28 d内58.0％（69/119）的感染是轻度至中度的，危及生命的感染很少发生（5.9％，7/119），且不同肿瘤感染的严重程度差异无统计学意义（感染例次数：*P*＝0.605；感染例数：*P*＝0.724）。

**表3:** 回输CAR-T细胞后28 d内不同感染类型的感染例次数、感染例数及感染密度

感染严重程度	感染例次数（例次）				感染例数［例（％）］			
	总体	ALL	NHL	MM	总体	ALL	NHL	MM
轻度	16	7	6	3	16（9.41）	7（4.12）	6（3.53）	3（1.76）
中度	53	28	12	13	42（24.7）	19（11.18）	12（7.10）	11（6.47）
重度	43	22	10	11	34（20.0）	16（9.41）	10（5.90）	8（4.71）
危及生命	7	4	1	2	7（4.12）	4（2.35）	1（0.59）	2（1.18）

*H*值	1.006				0.646			
*P*值	0.605				0.724			

注：CAR-T细胞：嵌合抗原受体T细胞；ALL：急性淋巴细胞白血病；NHL：非霍奇金淋巴瘤；MM：多发性骨髓瘤

119例次感染中，75次（63.0％）感染事件发生在粒缺期。90％血流感染的患者在发生感染时伴有中性粒细胞减少，而发生病毒感染和真菌感染的患者在粒缺期和非粒缺期分布比例相同。

78例患者既发生了CRS也发生了感染事件，不同等级CRS患者的感染风险曲线见图1，1～4级CRS的感染累积发生率分别为（52.6±8.1）％、（62.2±1.2）％、（98.8±6.5）％及（88.9±10.5）％。比较CRS发生时间和首次感染发生时间的感染风险曲线如图2所示，CRS的累积发生率为68.8％，感染的累积发生率为58.2％，CRS和首次感染的中位发生时间分别为7 d和12 d（*P*<0.001）。在3～4级CRS患者中，30次感染中有18次（60.0％）发生在CRS的峰值之后。

**图1:**
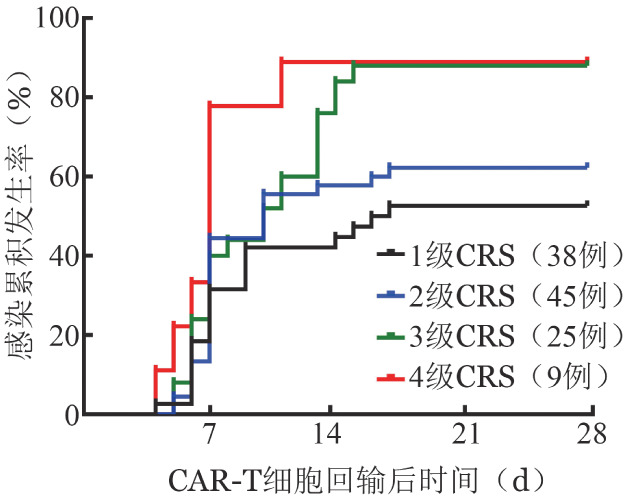
回输CAR-T细胞后发生不同等级细胞因子释放综合征（CRS）患者的感染发生曲线 CAR-T细胞：嵌合抗原受体T细胞

**图2:**
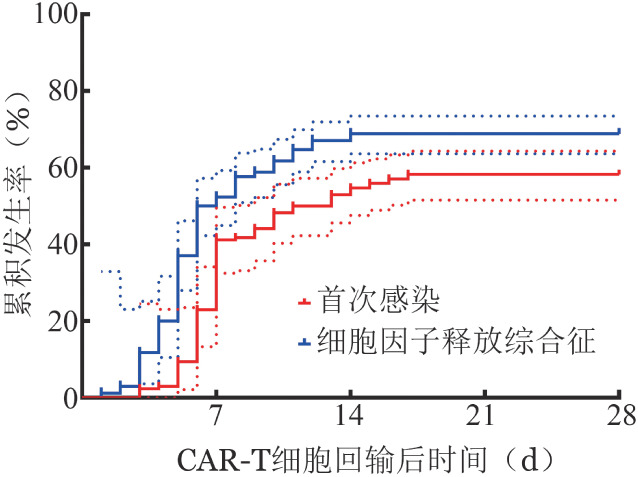
回输CAR-T细胞后CRS发生与首次感染的发生曲线比较

3. 基线特征与感染密度的关系：对发生感染的患者回输前基线特征进行单因素和多因素泊松回归，多因素模型结果显示，ALL患者、既往30 d感染病史、难治性疾病、前期治疗方案≥4种以及回输前ANC<0.5×10^9^/L与感染密度有更高的相关性（表4）。

**表4:** 泊松回归分析基线特征与CAR-T细胞回输后28 d内感染密度的关系

回输前基线指标	单因素分析		多因素分析	
	*IRR*（95％ *CI*）	*P*值	*IRR*（95％ *CI*）	*P*值
年龄	0.89（0.60～1.33）	0.58		
女性	0.97（0.66～1.43）	0.88		
有基础疾病	0.57（0.13～0.75）	0.98		
疾病类型				
ALL	参照		参照	
NHL	1.57（0.83～2.57）	0.04	4.12（1.85～6.43）	<0.001
MM	1.12（0.43～2.13）	0.21	2.78（1.93～4.67）	0.03
移植史	0.80（0.51～1.25）	0.33		
感染史^a^	1.25（0.37～3.08）	0.18	3.77（2.51～5.63）	<0.001
前期治疗方案≥4种	1.63（1.06～2.49）	0.02	3.12（1.31～4.01）	0.04
难治	1.50（1.01～2.23）	0.05	2.54（1.78～3.91）	<0.001
化疗前IgG<4mg/L	1.07（0.69～1.67）	0.77		
化疗前ALC<0.2×10^9^/L	0.76（0.50～1.15）	0.20		
化疗前ANC<0.5×10^9^/L	1.12（0.69～1.83）	0.64		
回输前ANC<0.5×10^9^/L	1.74（1.06～2.83）	0.03	2.11（0.75～3.02）	0.03
回输CAR-T细胞量				
2×10^6^/kg对4×10^6^/kg	0.70（0.48～1.52）	0.25		

注：ALL：急性淋巴细胞白血病；NHL：非霍奇金淋巴瘤；MM：多发性骨髓瘤；CAR-T细胞：嵌合抗原受体T细胞。a：既往30d内发生感染

4. 回输后感染的危险因素分析：单因素Cox回归显示，既往30 d内感染病史、感染发生时粒缺持续时间≥7 d、感染时ANC<0.5×10^9^/L、≥3级CRS、使用托珠单抗和进入ICU治疗与感染风险增加相关（表5），感染时ANC<0.5×10^9^/L发生感染风险为感染时ANC≥0.5×10^9^/L的1.86倍（95％ *CI* 1.22～2.84）；≥3级CRS和使用托珠单抗的感染风险分别增加3.58倍（95％*CI* 2.69～4.77）和3.84倍（95％*CI*2.48～5.93）。在多因素分析中，≥3级CRS是发生感染的独立危险因素（*HR*＝3.74，95％ *CI* 1.29～5.72，*P*<0.001）。

**表5:** 单因素Cox比例回归模型分析CAR-T细胞回输后28 d内感染的影响因素

因素	HR（95％CI）	*P*值
年龄	0.61（0.42～1.13）	0.23
女性	1.32（0.76～1.89）	0.10
有基础疾病	0.48（0.22～0.86）	0.79
疾病类型		
急性髓系白血病	参照	
非霍奇金淋巴瘤	2.21（1.35～4.23）	0.32
多发性骨髓瘤	1.75（0.65～2.33）	0.28
移植史	1.98（0.89～3.01）	0.19
感染史^a^	1.15（0.65～2.13）	0.01
前期治疗方案≥4种	1.36（0.48～1.85）	0.54
难治	1.56（0.89～2.67）	0.61
IgG<4mg/L	1.03（0.45～3.02）	0.10
ALC<0.2×10^9^/L	0.83（0.69～1.64）	0.42
ANC<0.5×10^9^/L	1.12（0.22～1.87）	0.33
ANC<0.5×10^9^/L	1.52（0.56～1.99）	0.15
回输CAR-T细胞量		
2×10^6^/kg对4×10^6^/kg	0.91（0.31～1.25）	0.71
白蛋白低于正常	1.24（0.38～1.74）	0.10
感染发生时粒缺持续时间≥7d	1.91（1.32～3.85）	0.04
感染时ANC<0.5×10^9^/L	1.86（1.22～2.84）	<0.01
≥3级CRS	3.58（2.69～4.77）	<0.01
神经毒性	1.73（0.76～3.96）	0.19
托珠单抗治疗	3.84（2.48～5.93）	<0.01
糖皮质激素治疗	1.83（1.15～2.91）	0.01
进入ICU治疗	1.02（0.66～2.35）	0.06

注：CRS：细胞因子释放综合征；ICU：重症监护室；a：既往30 d内发生感染

5. 严重感染与血清IL-6水平的二次升高：在发生严重感染的12例患者中，我们观察到有8例患者出现了IL-6水平的二次升高，且显著高于第一次升高的水平，这8例患者的共同特点是均为首次感染且感染均发生在CRS得到控制之后，而其他发生轻到重度感染和（或）发生CRS的患者中并没有出现这种现象。而在其余4例CRS与感染同时发生的患者，我们观察到这4例患者IL-6的水平只出现一个明显的高峰，且并没有显著高于单独发生CRS或感染时IL-6的水平（图3）。比较不同程度CRS和严重感染时的IL-6峰值，显示严重感染时IL-6水平高于发生3～4级CRS时的IL-6水平［1341（1134,1728）ng/L对915（725,1155）ng/L，*z*＝−3.364，*P*＝0.001］。其中1例严重感染患者血清IL-6水平>5 000 ng/L，而发生3～4级CRS患者中血清IL-6水平最高为1 556 ng/L。

**图3:**
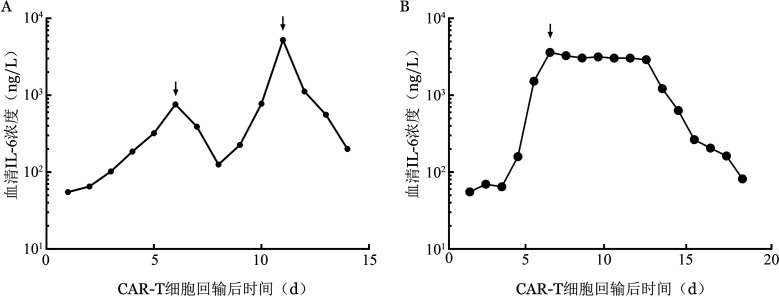
两例患者回输CAR-T细胞后血清IL-6浓度的动态变化（箭头表示血清IL-6峰浓度） CRS：细胞因子释放综合征；A：示CRS控制后发生严重感染患者IL-6水平的二次升高；B：示CRS与严重感染同时发生患者IL-6水平仅一个峰值

## 讨论

由于血液肿瘤患者经过前期多次化疗和本身的疾病状态，大多数接受CAR-T细胞治疗的患者处于免疫缺陷状态；回输CAR-T细胞前的淋巴耗竭方案也会引起全血细胞减少和黏膜屏障的损害[13]–[16]；而CAR-T细胞治疗的同时也会引起CRS和神经毒性，并且治疗上述并发症所需的托珠单抗和糖皮质激素均会增加感染的风险[17]；同时抗CD19 CAR-T细胞回输到人体内后会破坏正常的CD19^+^ B淋巴细胞，从而引起低免疫球蛋白血症[14]–[15]。这些因素综合引起了CAR-T治疗后感染风险的增加。本研究中，回输CAR-T细胞28 d内，感染的累积发生率为（58.2 ± 3.8）％。国外的一项临床试验（NCT01865617）入组了133例包括ALL、慢性淋巴细胞白血病（CLL）和NHL的患者，接受抗CD19 CAR-T后28 d内感染的累积发生率为23％[5]；一项研究发现26周岁以内的ALL患者回输后28 d内的感染的发生率为40.0％[18]；Wudhikarn等[6]报道回输后一年内的感染发生率为63.3％。以上都表明感染是CAR-T治疗后比较常见的事件。

我们发现ALL患者感染发生率高于MM和NHL患者，其他研究也发现ALL患者相对其他疾病更易感染[5]，并且大多数感染发生在CAR-T细胞回输后的早期。在泊松回归模型中，与感染密度相关的为ALL患者、难治性疾病、更多的先前抗肿瘤治疗方案以及回输前粒缺的患者，可能与免疫功能受损严重明显增加感染风险有关。不同肿瘤间的差异可能是因为罹患急性白血病的患者往往接受了更强烈的治疗，如强烈的化疗或移植，导致更长时间的粒缺，最终引起更高的感染发生率，尤其是难治性疾病患者，往往前期有过多线治疗方案。本研究中性粒细胞缺乏持续28 d时，累积感染率可达43.5％，国内的研究表明粒缺持续28 d时，发热发生率高达83.0％[9]。数据分析表明，粒细胞缺乏也可能增加了CAR-T细胞回输后的感染风险，但这项研究并不能排除淋巴耗竭方案强度对回输后感染风险的影响，因此需要进行进一步的研究[19]。在Cox回归模型中，我们分析了CAR-T细胞回输后的危险因素，严重CRS的发生是感染最重要的预测因子[20]–[21]。大多数感染发生在CRS发作后，可能与接受托珠单抗的患者增加了感染的风险有关。但目前仍不确定的是高细胞因子水平，免疫抑制干预或积极的支持治疗是否会导致严重CRS或神经毒性患者的感染风险增加[22]–[23]。我们还发现发生CRS时干预得当的患者发生威胁生命或致命的感染很少见[6],[18]。关于IL-6水平再次升高用于预测严重感染，目前已有研究提出“IL-6双峰模型”的诊断标准，可以用于临床上预测严重感染[23]；但当CRS与严重感染同时发生时，这一方法可能存在限制。

感染的类型主要以细菌为主，Freifeld等[24]的研究显示大多数感染是由患者化疗后感染的典型细菌引起。病毒感染主要以呼吸道病毒和乙肝病毒暴发为主；侵袭性真菌感染不常见，但在2.94％的患者中是感染的重要原因，所有发生真菌感染的患者均事先接受了移植和（或）发生了严重的CRS，并进行过免疫抑制治疗，因此对于高危患者，可以考虑采用更积极的真菌预防措施。积极筛查感染源，包括血培养、分泌物培养及高通量测序检查增加阳性率；根据细菌谱特点给予预防性抗感染治疗。在接受CD19 CAR-T细胞治疗的患者中，尚无标准化的抗生素预防方法，Hill等[5]的研究表明抗生素预防和免疫球蛋白补充的感染预防方法相对有效。

总之，接受CAR-T细胞治疗患者与接受挽救性化疗患者的感染发生率和感染类型相似[25]–[28]，而回输CAR-T细胞后28 d内发生感染的高危因素包括ALL患者、既往30天内感染病史、难治性疾病、回输前ANC<0.5×10^9^/L、既往接受过多线抗肿瘤方案以及发生了较高级别CRS（3级以上），即免疫功能受损严重并伴有重度CAR-T相关毒性的患者是感染的高危人群。除此之外，“IL-6双峰模型”也可以用于临床上预测严重感染。因此通过上述特征早期识别高危患者，并尽早预防对提高CAR-T疗法临床应用安全性具有重要临床意义。
